# Supplementary figures and images for: Physiological and metabolomic consequences of reduced expression of the Drosophila brummer triglyceride Lipase
Source: PLoS One. 2021 Sep 21;16(9):e0255198. doi: 10.1371/journal.pone.0255198 (PMC8454933; doi:10.1371/journal.pone.0255198)

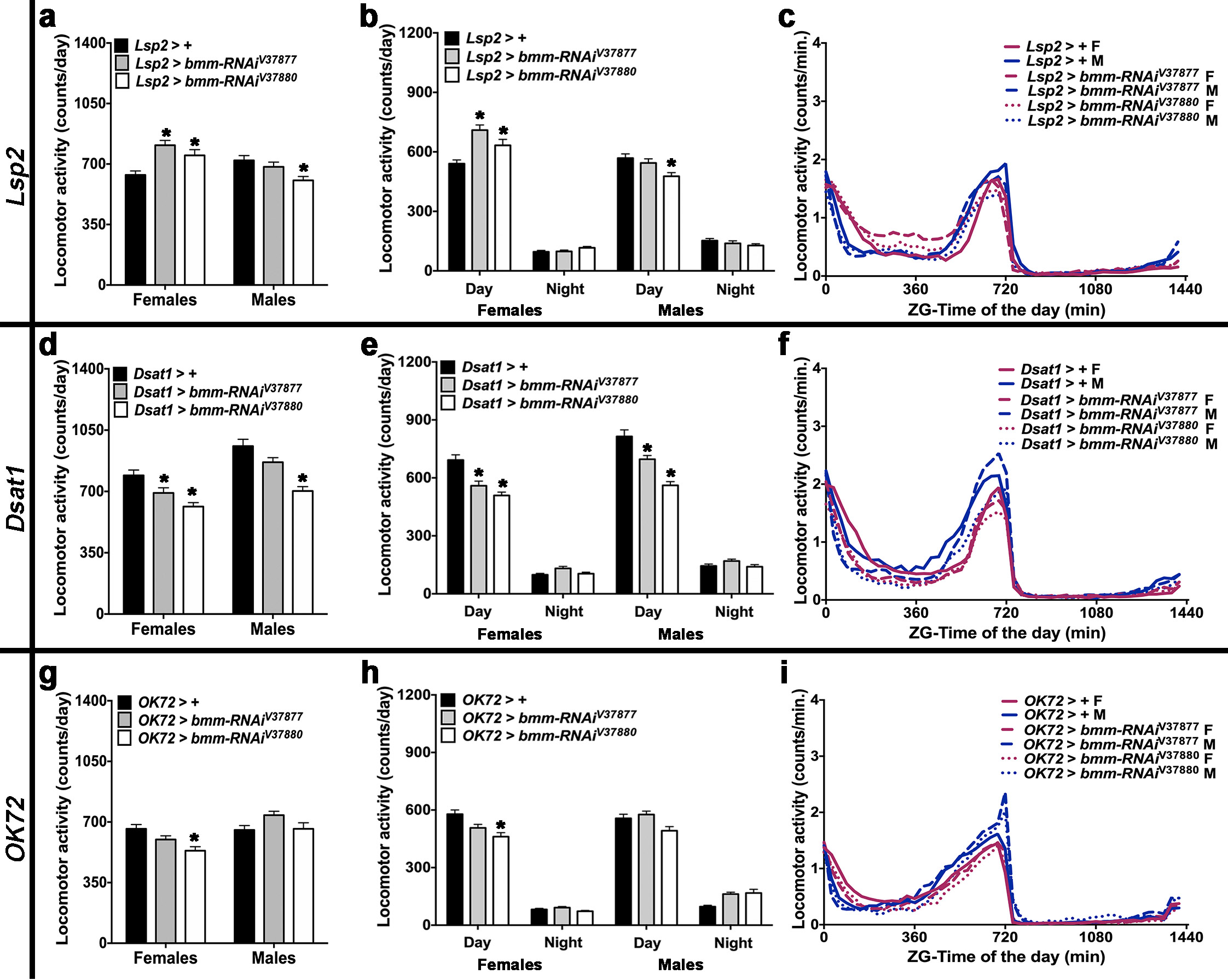

Supplement: S1 Fig — GD control and UAS-bmm-RNAi flies were mated with Lsp2-Gal4 (a-c), Dsat1-Gal4 (d-f) and OK72-Gal4 (g-i), and F1-flies were used for the assay in normal feeding. (a, d and g) Average of whole-day locomotor activity. (b, e and h) Average of locomotor activity during the daytime and nighttime. (c, f and i) Average activity profiles in Zeitgeber time. Averages were calculated from 7 days of behavior assay. For this and S2 Fig, “n” for females (F) and males (M) were: Lsp2 > + F (n = 60), Lsp2 > + M (n = 63), Lsp2 > bmm-RNAiV37877 F (n = 55), Lsp2 > bmm-RNAiV37877 M (n = 57), Lsp2 > bmm-RNAiV37880 F (n = 57), Lsp2 > bmm-RNAiV37880 M (n = 61), Dsat1 > + F (n = 62), Dsat1 > + M (n = 63), Dsat1 > bmm-RNAiV37877 F (n = 63), Dsat1 > bmm-RNAiV37877 M (n = 63), Dsat1 > bmm-RNAiV37880 F (n = 63), Dsat1 > bmm-RNAiV37880 M (n = 63), OK72 > + F (n = 58), OK72 > + M (n = 54), OK72 > bmm-RNAiV37877 F (n = 61), OK72 > bmm-RNAiV37877 M (n = 60), OK72 > bmm-RNAiV37880 F (n = 63) and OK72 > bmm-RNAiV37880 M (n = 61). Asterisks indicate significant differences at p < 0.05 compared with the appropriate female or male control, following Tukey’s correction for multiple tests. Error bars are SEM. ANOVA tests are reported in S4 Table. (JPG) [file pone.0255198.s001.jpg]

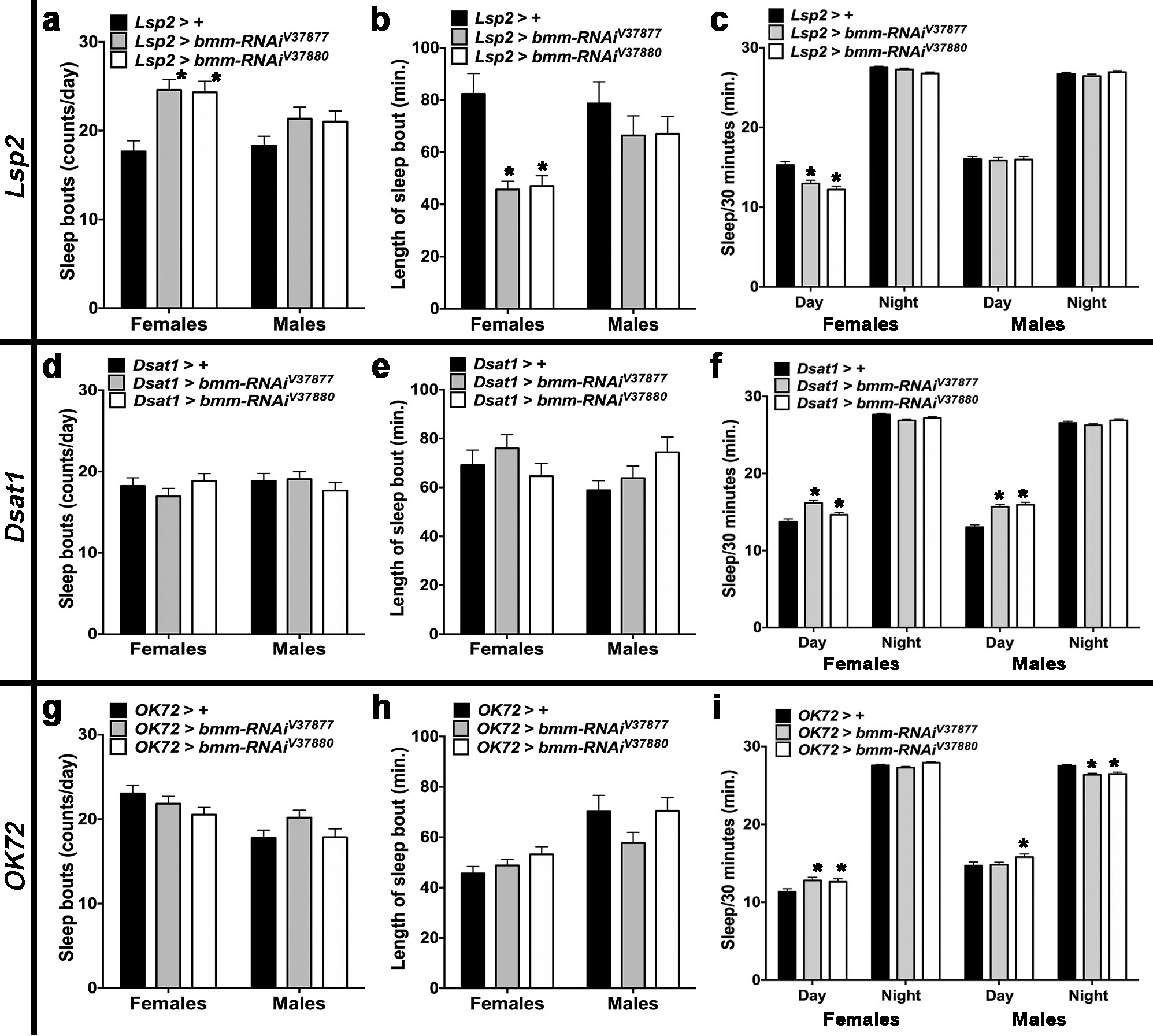

Supplement: S2 Fig — GD control and UAS-bmm-RNAi flies were mated with Lsp2-Gal4 (a-c), Dsat1-Gal4 (d-f) and OK72-Gal4 (g-i), and F1-flies were used for the assay in normal feeding. (a, d and g) Average number of sleep bouts. (b, e and h) Average length of sleep bout. (c, f and i) Average sleep during the daytime and the nighttime. Sleep was calculated using the standard definition of continuous period of inactivity lasting at least 5 minutes. Averages were calculated from 7 days of behavior assay and recalculated to periods of 30 minutes of sleep. Asterisks indicate significant differences at p < 0.05 compared with the appropriate female or male control, following Tukey’s correction for multiple tests. Error bars are SEM. ANOVA tests are reported in S5 Table. (JPG) [file pone.0255198.s002.jpg]

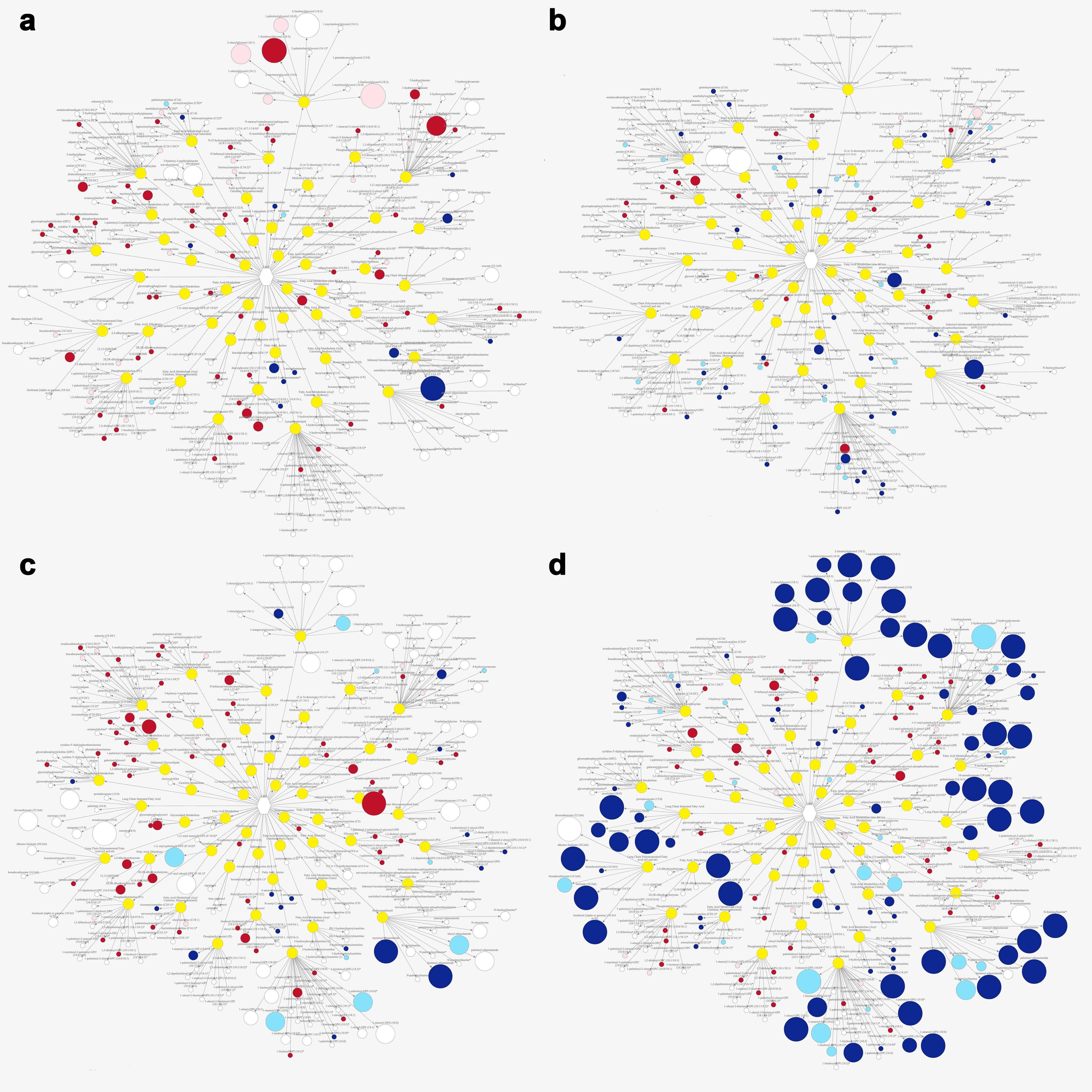

Supplement: S3 Fig — (a) Metabolites that significantly changed in Ubi > bmm-RNAiV37877 females compared with control females. (b) Metabolites that significantly changed in Ubi > bmm-RNAiV37880 females compared with control females. (c) Metabolites that significantly changed in Ubi > bmm-RNAiV37877 males compared with control males. (d) Metabolites that significantly changed in Ubi > bmm-RNAiV37880 males compared with control males. Yellow nodes represent the sub-pathways analyzed (see S1 Dataset). Red and dark blue represent the metabolites that increased and decreased respectively at p ≤ 0.05, light red and light blue represent the metabolites that increased and decreased respectively at 0.05 ≤ p ≤ 0.1, and size of circles represent the magnitude of change. ANOVA contrasts were used to identify metabolites that differed significantly between experimental groups and proper controls, and q-value is reported in S1 Dataset. (JPG) [file pone.0255198.s003.jpg]

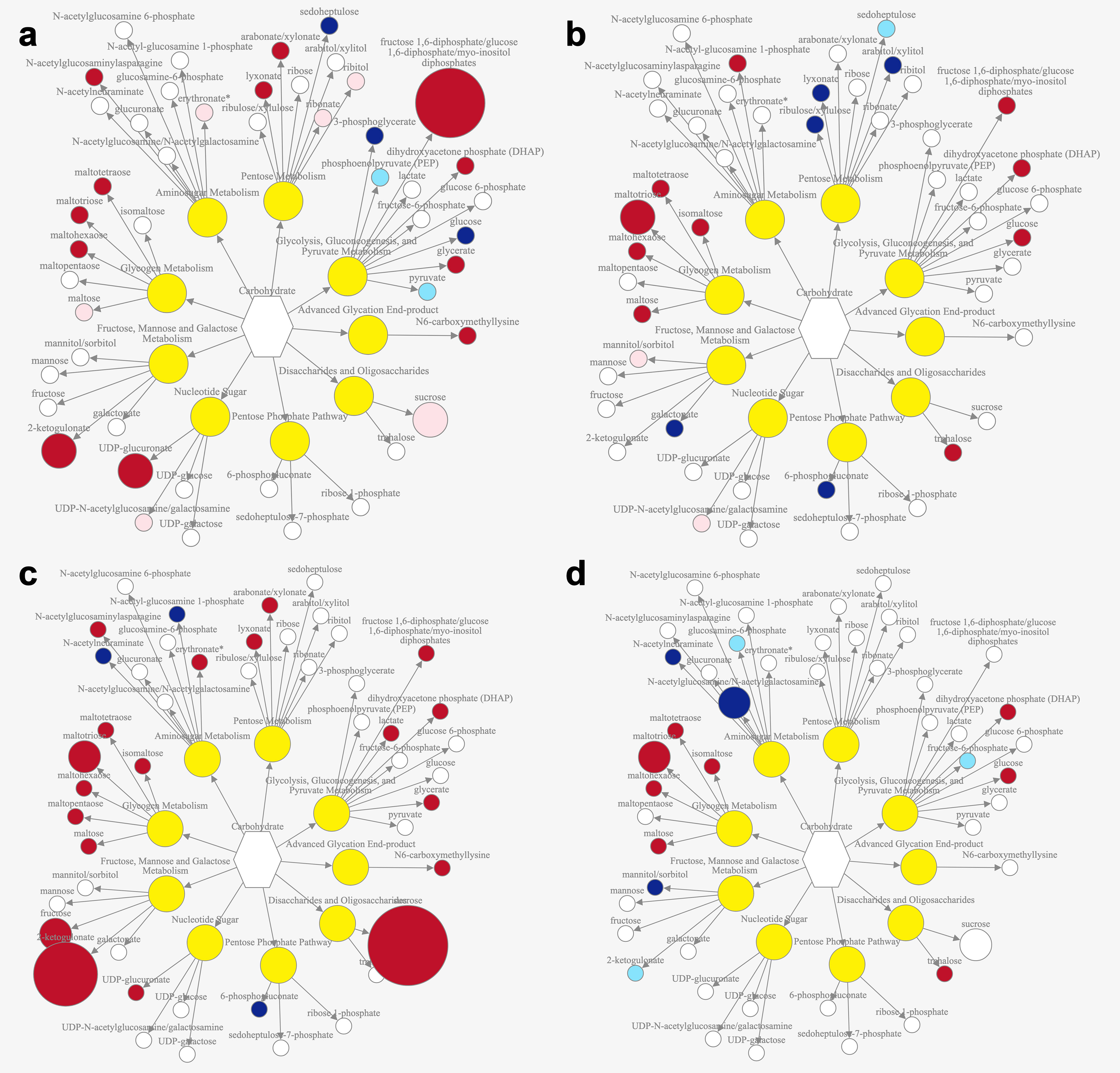

Supplement: S4 Fig — (a) Metabolites that significantly changed in Ubi > bmm-RNAiV37877 females compared with control females. (b) Metabolites that significantly changed in Ubi > bmm-RNAiV37880 females compared with control females. (c) Metabolites that significantly changed in Ubi > bmm-RNAiV37877 males compared with control males. (d) Metabolites that significantly changed in Ubi > bmm-RNAiV37880 males compared with control males. Yellow nodes represent the sub-pathways analyzed (see S1 Dataset). Red and dark blue represent the metabolites that increased and decreased respectively at p ≤ 0.05, light red and light blue represent the metabolites that increased and decreased respectively at 0.05 ≤ p ≤ 0.1, and size of circles represent the magnitude of change. ANOVA contrasts were used to identify metabolites that differed significantly between experimental groups and proper controls, and q-value is reported in S1 Dataset. (JPG) [file pone.0255198.s004.jpg]

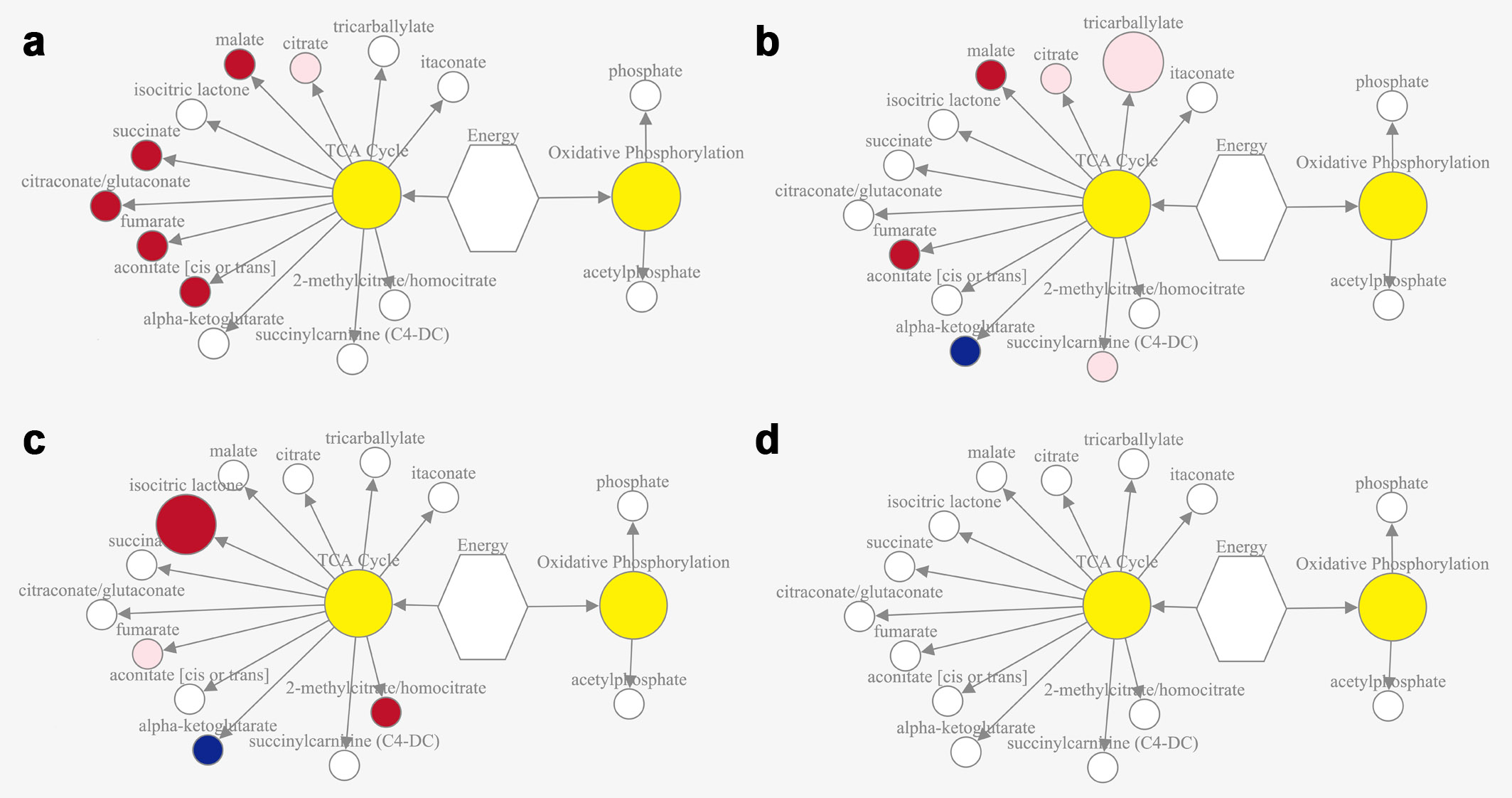

Supplement: S5 Fig — (a) Metabolites that significantly changed in Ubi > bmm-RNAiV37877 females compared with control females. (b) Metabolites that significantly changed in Ubi > bmm-RNAiV37880 females compared with control females. (c) Metabolites that significantly changed in Ubi > bmm-RNAiV37877 males compared with control males. (d) Metabolites that significantly changed in Ubi > bmm-RNAiV37880 males compared with control males. Yellow nodes represent the sub-pathways analyzed (see S1 Dataset). Red and dark blue represent the metabolites that increased and decreased respectively at p ≤ 0.05, light red and light blue represent the metabolites that increased and decreased respectively at 0.05 ≤ p ≤ 0.1, and size of circles represent the magnitude of change. ANOVA contrasts were used to identify metabolites that differed significantly between experimental groups and proper controls, and q-value is reported in S1 Dataset. (JPG) [file pone.0255198.s005.jpg]

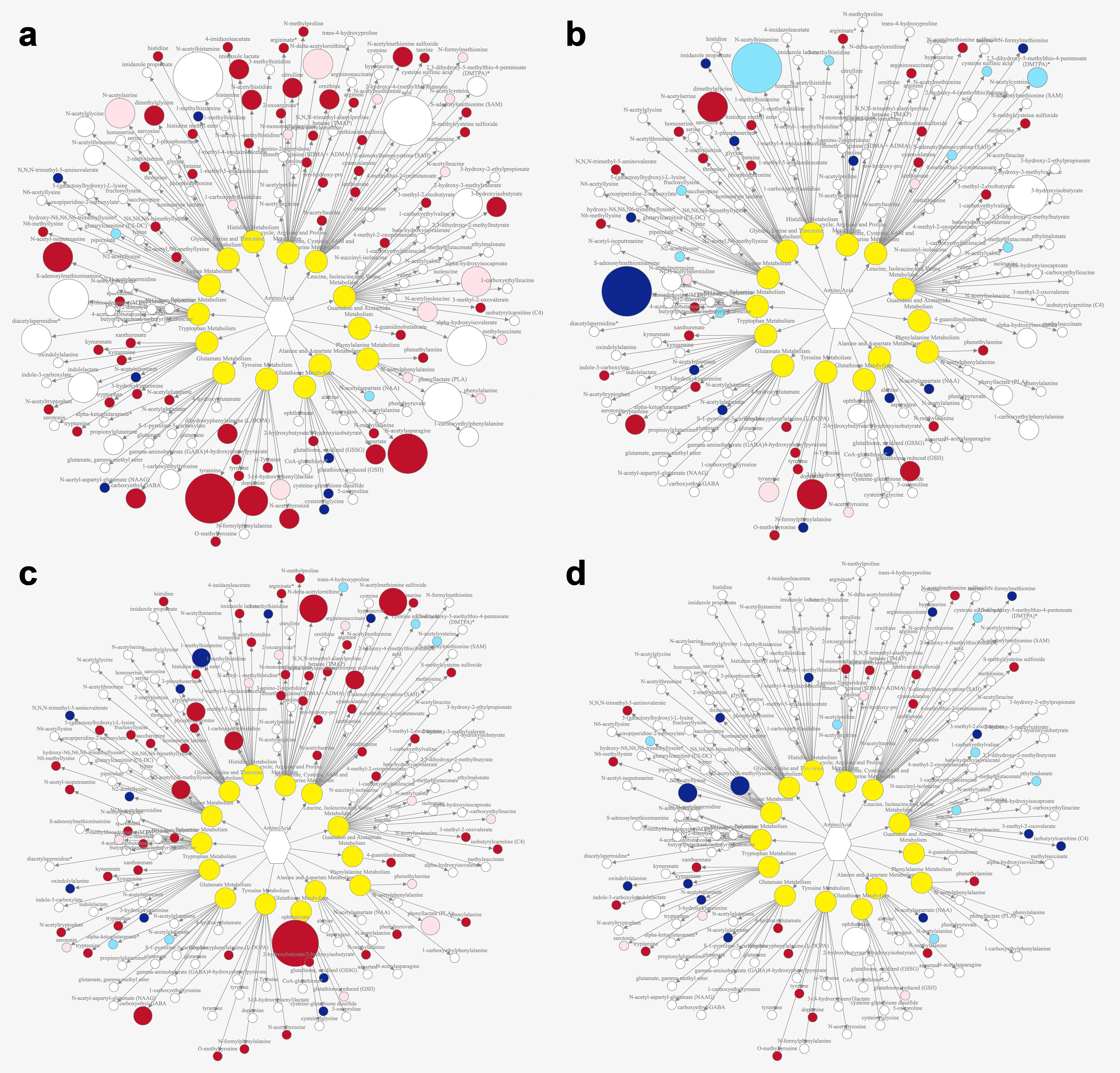

Supplement: S6 Fig — (a) Metabolites that significantly changed in Ubi > bmm-RNAiV37877 females compared with control females. (b) Metabolites that significantly changed in Ubi > bmm-RNAiV37880 females compared with control females. (c) Metabolites that significantly changed in Ubi > bmm-RNAiV37877 males compared with control males. (d) Metabolites that significantly changed in Ubi > bmm-RNAiV37880 males compared with control males. Yellow nodes represent the sub-pathways analyzed (see S1 Dataset). Red and dark blue represent the metabolites that increased and decreased respectively at p ≤ 0.05, light red and light blue represent the metabolites that increased and decreased respectively at 0.05 ≤ p ≤ 0.1, and size of circles represent the magnitude of change. ANOVA contrasts were used to identify metabolites that differed significantly between experimental groups and proper controls, and q-value is reported in S1 Dataset. (JPG) [file pone.0255198.s006.jpg]

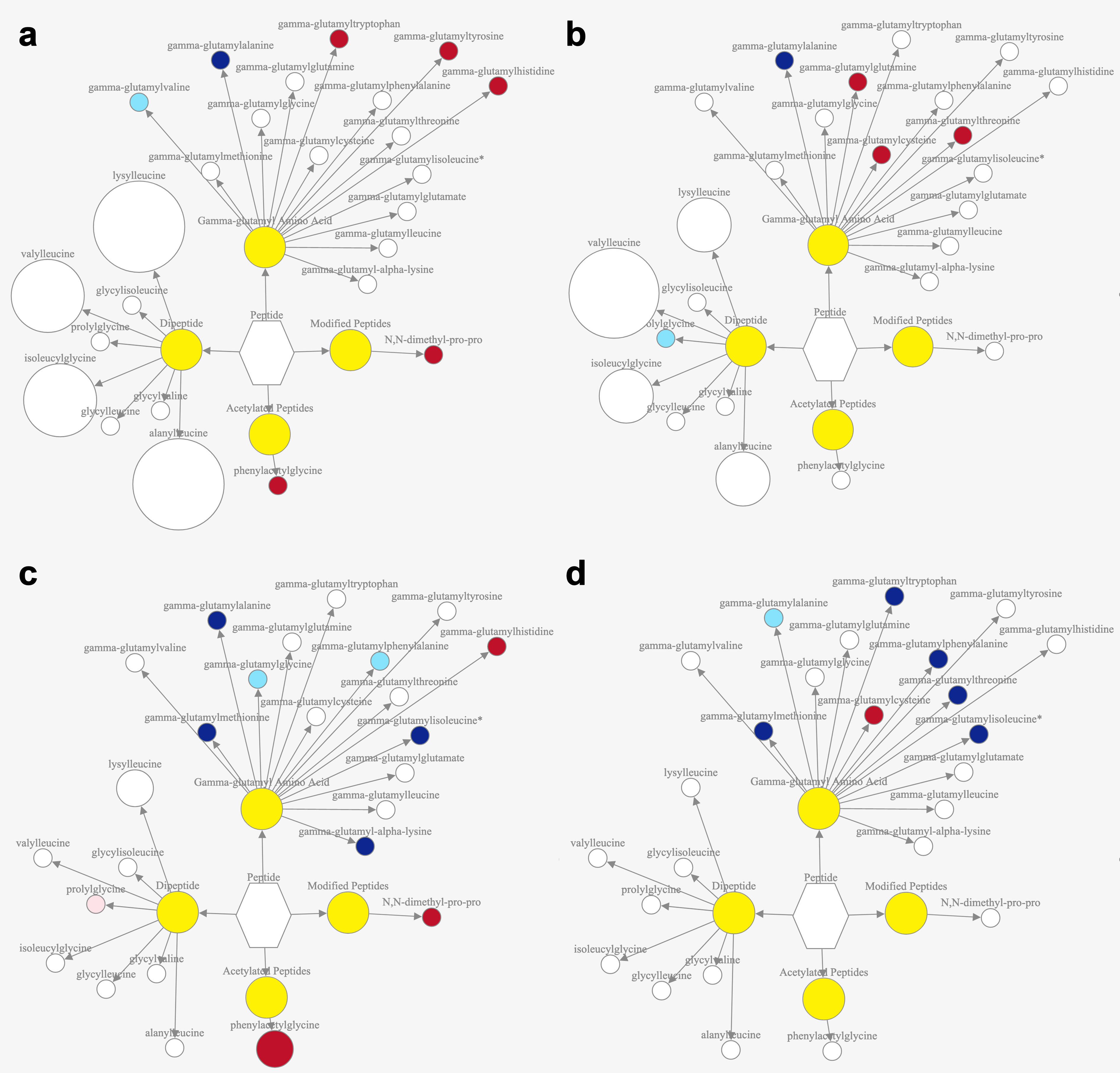

Supplement: S7 Fig — (a) Metabolites that significantly changed in Ubi > bmm-RNAiV37877 females compared with control females. (b) Metabolites that significantly changed in Ubi > bmm-RNAiV37880 females compared with control females. (c) Metabolites that significantly changed in Ubi > bmm-RNAiV37877 males compared with control males. (d) Metabolites that significantly changed in Ubi > bmm-RNAiV37880 males compared with control males. Yellow nodes represent the sub-pathways analyzed (see S1 Dataset). Red and dark blue represent the metabolites that increased and decreased respectively at p ≤ 0.05, light red and light blue represent the metabolites that increased and decreased respectively at 0.05 ≤ p ≤ 0.1, and size of circles represent the magnitude of change. ANOVA contrasts were used to identify metabolites that differed significantly between experimental groups and proper controls, and q-value is reported in S1 Dataset. (JPG) [file pone.0255198.s007.jpg]

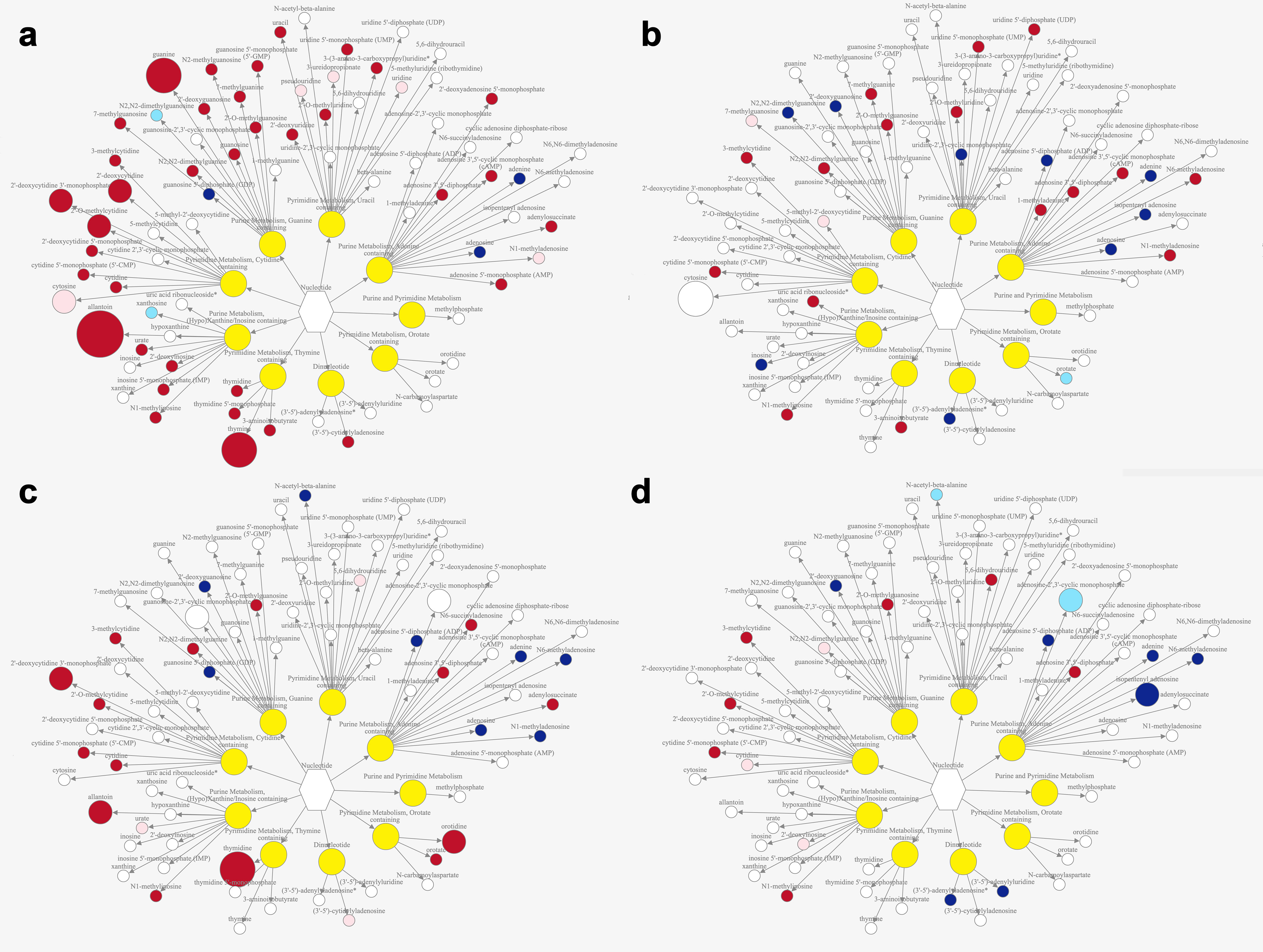

Supplement: S8 Fig — (a) Metabolites that significantly changed in Ubi > bmm-RNAiV37877 females compared with control females. (b) Metabolites that significantly changed in Ubi > bmm-RNAiV37880 females compared with control females. (c) Metabolites that significantly changed in Ubi > bmm-RNAiV37877 males compared with control males. (d) Metabolites that significantly changed in Ubi > bmm-RNAiV37880 males compared with control males. Yellow nodes represent the sub-pathways analyzed (see S1 Dataset). Red and dark blue represent the metabolites that increased and decreased respectively at p ≤ 0.05, light red and light blue represent the metabolites that increased and decreased respectively at 0.05 ≤ p ≤ 0.1, and size of circles represent the magnitude of change. ANOVA contrasts were used to identify metabolites that differed significantly between experimental groups and proper controls, and q-value is reported in S1 Dataset. (JPG) [file pone.0255198.s008.jpg]
