# Supplementary material for: Physiological and metabolomic consequences of reduced expression of the Drosophila brummer triglyceride Lipase
Source: PLoS One. 2021 Sep 21;16(9):e0255198. doi: 10.1371/journal.pone.0255198 (PMC8454933; doi:10.1371/journal.pone.0255198)
Supplement: S1 Table — (PDF) [file pone.0255198.s009.pdf]

Table S1. ANOVA of RT-qPCR and lifespan of *Ubi > bmm-RNAi* flies.

**RT-qPCR**						
	**Df**	**Sum-Sq**	**Mean-Sq**	**F-value**	**Pr (>F)**	**Signif.**
**Genotype**	2	0.04132	0.02066	83.54	p <0.0001	***
**Sex**	1	0.003977	0.003977	16.08	0.0002	***
**Interaction**	2	0.0002198	0.0001099	0.4444	0.6438	ns
**Residuals**	48	0.01187	0.0002473			
**Lifespan**						
	**Df**	**Sum-Sq**	**Mean-Sq**	**F-value**	**Pr (>F)**	**Signif.**
**Genotype**	2	79291.59052	39645.79526	265.57	p <0.0001	***
**Sex**	1	5494.71991	5494.71991	36.81	p <0.0001	***
**Vial(Genotype)**	144	66070.79284	458.82495	3.07	p <0.0001	***
**Vial*Sex(Genotype)**	146	65326.97594	447.44504	3.00	p <0.0001	***
**Residuals**	495	73896.1667	149.2852			

ns=not significant, * $p < 0.05$, ** $p < 0.01$, *** $p < 0.001$.

For RT-qPCR n=9, “n” was equal in three genotypes and between sexes.

For lifespan “n” were: *Ubi* > + (n=132 for females and 118 for males), *Ubi* > *bmm-RNAi*^{V37877} (n=136 for females and 134 for males) and *Ubi* > *bmm-RNAi*^{V37880} (n=137 for females and 132 for males).
