# Supplementary material for: Physiological and metabolomic consequences of reduced expression of the Drosophila brummer triglyceride Lipase
Source: PLoS One. 2021 Sep 21;16(9):e0255198. doi: 10.1371/journal.pone.0255198 (PMC8454933; doi:10.1371/journal.pone.0255198)
Supplement: S2 Table — (PDF) [file pone.0255198.s010.pdf]

Table S2. ANOVAs of locomotor activity of *Ubi > bmm-RNAi* flies.

**Whole-day locomotor activity in normal feeding.**						
	**Df**	**Sum-Sq**	**Mean-Sq**	**F-value**	**Pr (>F)**	**Signif.**
**Genotype**	2	1.662e+006	830838	6.545	0.0016	**
**Sex**	1	8065	8065	0.06354	0.8012	ns
**Interaction**	2	4.441e+006	2.220e+006	17.49	p < 0.0001	***
**Residuals**	326	4.138e+007	126938			
**Whole-day locomotor activity in normal feeding without wings.**						
	**Df**	**Sum-Sq**	**Mean-Sq**	**F-value**	**Pr (>F)**	**Signif.**
**Genotype**	2	3.269e+006	1.634e+006	55.56	p <0.0001	***
**Sex**	1	105992	105992	3.603	0.0585	ns
**Interaction**	2	1.060e+006	529770	18.01	p <0.0001	***
**Residuals**	350	1.030e+007	29415			
**Whole-day locomotor activity in Starvation.**						
	**Df**	**Sum-Sq**	**Mean-Sq**	**F-value**	**Pr (>F)**	**Signif.**
**Genotype**	2	1.868e+006	934021	7.777	0.0005	***
**Sex**	1	815712	815712	6.792	0.0096	**
**Interaction**	2	1.646e+007	8.228e+006	68.51	p <0.0001	***
**Residuals**	324	3.891e+007	120095			

Locomotor activity during the daytime and nighttime in normal feeding.						
	**Df**	**Sum-Sq**	**Mean-Sq**	**F-value**	**Pr (>F)**	**Signif.**
**Genotype**	2	830838	415419	8.127	0.0003	***
**Sex(Time)**	3	2.444e+007	8.145e+006	159.4	p < 0.0001	***
**Interaction**	6	3.188e+006	531269	10.39	p < 0.0001	***
**Residuals**	652	3.333e+007	51117			
**Locomotor activity during the daytime and nighttime in normal feeding without wings.**						
	**Df**	**Sum-Sq**	**Mean-Sq**	**F-value**	**Pr (>F)**	**Signif.**
**Genotype**	2	1.634e+006	817125	66.05	p <0.0001	***
**Sex(Time)**	3	3.020e+007	1.007e+007	813.6	p <0.0001	***
**Interaction**	6	1.379e+006	229784	18.57	p <0.0001	***
**Residuals**	700	8.660e+006	12371			
**Locomotor activity during the daytime and nighttime in starvation.**						
	**Df**	**Sum-Sq**	**Mean-Sq**	**F-value**	**Pr (>F)**	**Signif.**
**Genotype**	2	933806	466903	10.31	p <0.0001	***
**Sex(Time)**	3	7.462e+007	2.487e+007	549.3	p <0.0001	***
**Interaction**	6	1.187e+007	1.978e+006	43.69	p <0.0001	***
**Residuals**	648	2.934e+007	45278			

ns=not significant, * p < 0.05, ** p < 0.01, *** p < 0.001.

"n" in normal feeding were: *Ubi* > + F (n=49), *Ubi* > + M (n=57), *Ubi > bmm-RNAi*^{V37877} F (n=54), *Ubi > bmm-RNAi*^{V37877} M (n=53), *Ubi > bmm-RNAi*^{V37880} F (n=57) and *Ubi > bmm-RNAi*^{V37880} M (n=62).

"n" in normal feeding without wings were: *Ubi* > + F (n=64), *Ubi* > + M (n=62), *Ubi > bmm-RNAi*^{V37877} F (n=56), *Ubi > bmm-RNAi*^{V37877} M (n=55), *Ubi > bmm-RNAi*^{V37880} F (n=58) and *Ubi > bmm-RNAi*^{V37880} M (n=61).

"n" in starvation were: *Ubi* > + F (n=56), *Ubi* > + M (n=42), *Ubi > bmm-RNAi*^{V37877} F (n=54), *Ubi > bmm-RNAi*^{V37877} M (n=52), *Ubi > bmm-RNAi*^{V37880} F (n=63) and *Ubi > bmm-RNAi*^{V37880} M (n=63).
