# Supplementary material for: Physiological and metabolomic consequences of reduced expression of the Drosophila brummer triglyceride Lipase
Source: PLoS One. 2021 Sep 21;16(9):e0255198. doi: 10.1371/journal.pone.0255198 (PMC8454933; doi:10.1371/journal.pone.0255198)
Supplement: S3 Table — (PDF) [file pone.0255198.s011.pdf]

Table S3. ANOVAs of sleep of *Ubi > bmm-RNAi* flies.

**Sleep bouts in normal feeding**						
	**Df**	**Sum-Sq**	**Mean-Sq**	**F-value**	**Pr (>F)**	**Signif.**
**Genotype**	2	626.7	313.3	3.646	0.0272	*
**Sex**	1	457.1	457.1	5.319	0.0217	*
**Interaction**	2	2846	1423	16.56	p <0.0001	***
**Residuals**	326	28016	85.94			
**Sleep bouts in normal feeding without wings**						
	**Df**	**Sum-Sq**	**Mean-Sq**	**F-value**	**Pr (>F)**	**Signif.**
**Genotype**	2	159.7	79.83	1.284	0.2783	ns
**Sex**	1	227.4	227.4	3.656	0.0567	ns
**Interaction**	2	866.5	433.3	6.967	0.0011	**
**Residuals**	350	21766	62.19			
**Sleep bouts in starvation**						
	**Df**	**Sum-Sq**	**Mean-Sq**	**F-value**	**Pr (>F)**	**Signif.**

**Genotype**	2	2649	1324	19.54	p <0.0001	***
**Sex**	1	351.9	351.9	5.192	0.0233	*
**Interaction**	2	970.8	485.4	7.161	0.0009	***
**Residuals**	324	21960	67.78			
**Sleep bout lengths in normal feeding**						
	**Df**	**Sum-Sq**	**Mean-Sq**	**F-value**	**Pr (>F)**	**Signif.**
**Genotype**	2	3748	1874	2.583	0.0771	ns
**Sex**	1	8.540	8.540	0.01177	0.9137	ns
**Interaction**	2	18473	9236	12.73	p <0.0001	***
**Residuals**	326	236488	725.4			
**Sleep bout lengths in normal feeding without wings**						
	**Df**	**Sum-Sq**	**Mean-Sq**	**F-value**	**Pr (>F)**	**Signif.**
**Genotype**	2	6231	3115	3.988	0.0194	*
**Sex**	1	2980	2980	3.814	0.0516	ns
**Interaction**	2	9840	4920	6.298	0.0021	**
**Residuals**	350	273443	781.3			
**Sleep bout lengths in starvation**						
	**Df**	**Sum-Sq**	**Mean-Sq**	**F-value**	**Pr (>F)**	**Signif.**
**Genotype**	2	29032	14516	8.949	0.0002	***
**Sex**	1	5390	5390	3.323	0.0692	ns
**Interaction**	2	9942	4971	3.065	0.0480	*
**Residuals**	324	525541	1622			
**Sleep during the daytime and the nighttime in normal feeding**						
	**Df**	**Sum-Sq**	**Mean-Sq**	**F-value**	**Pr (>F)**	**Signif.**
**Genotype**	2	1.066	0.5330	60.38	p <0.0001	***
**Sex**	3	21.17	7.055	799.2	p <0.0001	***
**Interaction**	6	1.065	0.1774	20.10	p <0.0001	***
**Residuals**	652	5.755	0.008827			
**Sleep during the daytime and the nighttime in normal feeding without wings**						
	**Df**	**Sum-Sq**	**Mean-Sq**	**F-value**	**Pr (>F)**	**Signif.**
**Genotype**	2	1.066	0.5329	86.22	p <0.0001	***
**Sex**	3	34.98	11.66	1886	p <0.0001	***
**Interaction**	6	0.8239	0.1373	22.22	p <0.0001	***
**Residuals**	818	5.056	0.006181			
**Sleep during the daytime and the nighttime in starvation**						
	**Df**	**Sum-Sq**	**Mean-Sq**	**F-value**	**Pr (>F)**	**Signif.**
**Genotype**	2	0.2481	0.1241	11.15	p <0.0001	***
**Sex**	3	41.97	13.99	1258	p <0.0001	***
**Interaction**	6	2.843	0.4738	42.60	p <0.0001	***
**Residuals**	648	7.207	0.01112			

ns=not significant, * $p < 0.05$, ** $p < 0.01$, *** $p < 0.001$.

“n” in normal feeding were: *Ubi* > + F (n=49), *Ubi* > + M (n=57), *Ubi > bmm-RNAi*^{V37877} F (n=54), *Ubi > bmm-RNAi*^{V37877} M (n=53), *Ubi > bmm-RNAi*^{V37880} F (n=57) and *Ubi > bmm-RNAi*^{V37880} M (n=62).

“n” in normal feeding without wings were: *Ubi* > + F (n=64), *Ubi* > + M (n=62), *Ubi > bmm-RNAi*^{V37877} F (n=56), *Ubi > bmm-RNAi*^{V37877} M (n=55), *Ubi > bmm-RNAi*^{V37880} F (n=58) and *Ubi > bmm-RNAi*^{V37880} M (n=61).

“n” in starvation were: *Ubi* > + F (n=56), *Ubi* > + M (n=42), *Ubi > bmm-RNAi*^{V37877} F (n=54), *Ubi > bmm-RNAi*^{V37877} M (n=52), *Ubi > bmm-RNAi*^{V37880} F (n=63) and *Ubi > bmm-RNAi*^{V37880} M (n=63).
