# Supplementary material for: Physiological and metabolomic consequences of reduced expression of the Drosophila brummer triglyceride Lipase
Source: PLoS One. 2021 Sep 21;16(9):e0255198. doi: 10.1371/journal.pone.0255198 (PMC8454933; doi:10.1371/journal.pone.0255198)
Supplement: S4 Table — (PDF) [file pone.0255198.s012.pdf]

Table S4. ANOVAs of locomotor activity of *bmm-RNAi* lines expressed in fat-body (*Lsp2-Gal4*) and oenocytes (*Dsat1-Gal4* and *OK72-Gal4*) in normal feeding.

**Whole day locomotor activity of *Lsp2 > bmm-RNAi***						
	**Df**	**Sum-Sq**	**Mean-Sq**	**F-value**	**Pr (>F)**	**Signif.**
**Genotype**	2	357111	178556	4.160	0.0164	*
**Sex**	1	335810	335810	7.823	0.0054	**
**Interaction**	2	942262	471131	10.98	p <0.0001	***
**Residuals**	347	1.489e+007	42924			
**Whole day locomotor activity of *Dsat1 > bmm-RNAi***						
	**Df**	**Sum-Sq**	**Mean-Sq**	**F-value**	**Pr (>F)**	**Signif.**
**Genotype**	2	2.970e+006	1.485e+006	27.81	p <0.0001	***
**Sex**	1	1.952e+006	1.952e+006	36.55	p <0.0001	***
**Interaction**	2	145717	72858	1.365	0.2568	ns
**Residuals**	371	1.981e+007	53391			
**Whole day locomotor activity of *OK72 > bmm-RNAi***						
	**Df**	**Sum-Sq**	**Mean-Sq**	**F-value**	**Pr (>F)**	**Signif.**
**Genotype**	2	351829	175914	4.378	0.0132	*
**Sex**	1	666949	666949	16.60	p <0.0001	***
**Interaction**	2	390234	195117	4.856	0.0083	**
**Residuals**	351	1.410e+007	40177			
**Locomotor activity during the daytime and nighttime of *Lsp2 > bmm-RNAi***						
	**Df**	**Sum-Sq**	**Mean-Sq**	**F-value**	**Pr (>F)**	**Signif.**
**Genotype**	2	178555	89278	5.098	0.0063	**
**Sex(Time)**	3	3.777e+007	1.259e+007	719.0	p <0.0001	***
**Interaction**	6	960431	160072	9.140	p <0.0001	***
**Residuals**	694	1.215e+007	17513			
**Locomotor activity during the daytime and nighttime of *Dsat1 > bmm-RNAi***						
	**Df**	**Sum-Sq**	**Mean-Sq**	**F-value**	**Pr (>F)**	**Signif.**
**Genotype**	2	1.485e+006	742489	35.60	p <0.0001	***
**Sex(Time)**	3	4.972e+007	1.657e+007	794.8.4	p <0.0001	***
**Interaction**	6	1.721e+006	286847	13.76	p <0.0001	***
**Residuals**	742	1.547e+007	20854			
**Locomotor activity during the daytime and nighttime of *OK72 > bmm-RNAi***						
	**Df**	**Sum-Sq**	**Mean-Sq**	**F-value**	**Pr (>F)**	**Signif.**
**Genotype**	2	175915	87957	5.734	0.0034	***
**Sex(Time)**	3	3.130e+007	1.043e+007	680.2	p <0.0001	***
**Interaction**	6	666961	111160	7.247	p <0.0001	***
**Residuals**	702	1.077e+007	15339			

ns=not significant, * $p < 0.05$, ** $p < 0.01$, *** $p < 0.001$.

Lsp2 > + F (n=60), *Lsp2* > + M (n=63), *Lsp2 > bmm-RNAi*^{V37877} F (n=55), *Lsp2 > bmm-RNAi*^{V37877} M (n=57), *Lsp2 > bmm-RNAi*^{V37880} F (n=57) and *Lsp2 > bmm-RNAi*^{V37880} M (n=61).

Dsat1 > + F (n=62), *Dsat1* > + M (n=63), *Dsat1 > bmm-RNAi*^{V37877} F (n=63), *Dsat1 > bmm-RNAi*^{V37877} M (n=63), *Dsat1 > bmm-RNAi*^{V37880} F (n=63) and *Dsat1 > bmm-RNAi*^{V37880} M (n=63).

OK72 > + F (n=58), *OK72* > + M (n=54), *OK72 > bmm-RNAi*^{V37877} F (n=61), *OK72 > bmm-RNAi*^{V37877} M (n=60), *OK72 > bmm-RNAi*^{V37880} F (n=63) and *OK72 > bmm-RNAi*^{V37880} M (n=61).
