# Supplementary material for: Physiological and metabolomic consequences of reduced expression of the Drosophila brummer triglyceride Lipase
Source: PLoS One. 2021 Sep 21;16(9):e0255198. doi: 10.1371/journal.pone.0255198 (PMC8454933; doi:10.1371/journal.pone.0255198)
Supplement: S5 Table — (PDF) [file pone.0255198.s013.pdf]

Table S5. ANOVAs of sleep of *bmm-RNAi* lines expressed in fat-body (*Lsp2-Gal4*) and oenocytes (*Dsat1-Gal4* and *OK72-Gal4*) in normal feeding.

**Sleep bouts of *Lsp2 > bmm-RNAi***						
	**Df**	**Sum-Sq**	**Mean-Sq**	**F-value**	**Pr (>F)**	**Signif.**
**Genotype**	2	1842	921.2	10.84	p <0.0001	***
**Sex**	1	338.3	338.3	3.982	0.0468	*
**Interaction**	2	299.2	149.6	1.761	0.1734	ns
**Residuals**	347	29480	84.96			
**Sleep bouts of *Dsat1 > bmm-RNAi***						
	**Df**	**Sum-Sq**	**Mean-Sq**	**F-value**	**Pr (>F)**	**Signif.**
**Genotype**	2	18.76	9.380	0.1657	0.8474	ns
**Sex**	1	25.43	25.43	0.4493	0.5031	ns
**Interaction**	2	174.8	87.42	1.544	0.2148	ns
**Residuals**	371	21001	56.61			
**Sleep bouts of *OK72 > bmm-RNAi***						
	**Df**	**Sum-Sq**	**Mean-Sq**	**F-value**	**Pr (>F)**	**Signif.**
**Genotype**	2	199.4	99.72	1.987	0.1387	ns
**Sex**	1	907.5	907.5	18.08	p < 0.0001	***
**Interaction**	2	204.7	102.3	2.039	0.1317	ns
**Residuals**	351	17615	50.19			
**Sleep bout lengths of *Lsp2 > bmm-RNAi***						
	**Df**	**Sum-Sq**	**Mean-Sq**	**F-value**	**Pr (>F)**	**Signif.**
**Genotype**	2	44949	22475	8.499	0.0002	***
**Sex**	1	13434	13434	5.080	0.0248	*
**Interaction**	2	11241	5621	2.125	0.1209	ns
**Residuals**	347	917630	2644			
**Sleep bout lengths of *Dsat1 > bmm-RNAi***						
	**Df**	**Sum-Sq**	**Mean-Sq**	**F-value**	**Pr (>F)**	**Signif.**
**Genotype**	2	2729	1365	0.7463	0.4748	ns

	Df	Sum-Sq	Mean-Sq	F-value	Pr (>F)	Signif.
**Sex**	1	1668	1668	0.9121	0.3402	ns
**Interaction**	2	9285	4643	2.539	0.0803	ns
**Residuals**	371	678366	1828			
**Sleep bout lengths of *OK72 > bmm-RNAi***						
	**Df**	**Sum-Sq**	**Mean-Sq**	**F-value**	**Pr (>F)**	**Signif.**
**Genotype**	2	4413	2207	2.157	0.1172	ns
**Sex**	1	25647	25647	25.07	p < 0.0001	***
**Interaction**	2	3754	1877	1.834	0.1613	ns
**Residuals**	351	359126	1023			
**Sleep during the daytime and the nighttime of *Lsp2 > bmm-RNAi***						
	**Df**	**Sum-Sq**	**Mean-Sq**	**F-value**	**Pr (>F)**	**Signif.**
**Genotype**	2	111.9	55.94	9.231	0.0001	***
**Sex(Time)**	3	26844	8948	1476	p <0.0001	***
**Interaction**	6	215.9	35.99	5.938	p <0.0001	***
**Residuals**	694	4206	6.060			
**Sleep during the daytime and the nighttime of *Dsat1 > bmm-RNAi***						
	**Df**	**Sum-Sq**	**Mean-Sq**	**F-value**	**Pr (>F)**	**Signif.**
**Genotype**	2	156.6	78.29	17.64	p < 0.0001	***
**Sex(Time)**	3	27315	9105	2052	p < 0.0001	***
**Interaction**	6	391.9	65.31	14.72	p < 0.0001	***
**Residuals**	742	3293	4.438			
**Sleep during the daytime and the nighttime of *OK72 > bmm-RNAi***						
	**Df**	**Sum-Sq**	**Mean-Sq**	**F-value**	**Pr (>F)**	**Signif.**
**Genotype**	2	26.23	13.11	2.396	0.0919	ns
**Sex(Time)**	3	33276	11092	2026	p < 0.0001	***
**Interaction**	6	154.3	25.72	4.698	0.0001	***
**Residuals**	702	3843	5.474			

ns=not significant, * p < 0.05, ** p < 0.01, *** p < 0.001.

Lsp2 > + F (n=60), *Lsp2* > + M (n=63), *Lsp2 > bmm-RNAi*^{V37877} F (n=55), *Lsp2 > bmm-RNAi*^{V37877} M (n=57), *Lsp2 > bmm-RNAi*^{V37880} F (n=57) and *Lsp2 > bmm-RNAi*^{V37880} M (n=61).

Dsat1 > + F (n=62), *Dsat1* > + M (n=63), *Dsat1 > bmm-RNAi*^{V37877} F (n=63), *Dsat1 > bmm-RNAi*^{V37877} M (n=63), *Dsat1 > bmm-RNAi*^{V37880} F (n=63) and *Dsat1 > bmm-RNAi*^{V37880} M (n=63).

OK72 > + F (n=58), *OK72* > + M (n=54), *OK72 > bmm-RNAi*^{V37877} F (n=61), *OK72 > bmm-RNAi*^{V37877} M (n=60), *OK72 > bmm-RNAi*^{V37880} F (n=63) and *OK72 > bmm-RNAi*^{V37880} M (n=61).
